# Prevalence, distribution and factors associated with modern contraceptive use among women of reproductive age in Uganda: evidence from UDHS 2016

**DOI:** 10.1186/s40834-024-00288-6

**Published:** 2024-06-28

**Authors:** Moses Festo Towongo, Matlhogonolo Kelepile

**Affiliations:** 1 Department of Population Studies, University of Botswana, Gaborone, Botswana; 2 Department of Environmental Science, University of Botswana, Gaborone, Botswana

**Keywords:** Prevalence, Spatial distribution, Modern contraception, Utilization, Women, Uganda

## Abstract

**Background:**

Unintended pregnancies pose significant health risks, particularly in sub-Saharan Africa, where millions of cases are recorded annually, disproportionately affecting adolescent women. Utilization of modern contraceptives is crucial in managing fertility and reducing unintended pregnancies, abortions, and associated health complications. This study aimed to assess the prevalence, distribution and factors associated with modern contraceptives utilization among women aged 15–49 in Uganda.

**Methods:**

The study used secondary data from the 2016 Uganda Demographic and Health Survey (UDHS). The study sample comprise of 9,235 women aged 15–49 who used any method to prevent pregnancy in the five years preceding 2016 UDHS survey. The outcome variable for this study is utilization of modern contraceptives. Univariate, bivariate, and multilevel binary logistic regression was used to examine the relationship between individual and contextual factors on the modern contraceptive use among women aged 15–49 in Uganda. Choropleth mapping and network analysis in ArcGIS 10.8.2 was used to visualize spatial distribution of modern contraceptive use and measure community access to health facilities respectively.

**Results:**

The prevalence of modern contraceptive use was 53.19% (*n* = 4,919) in Uganda, with significant spatial variation by district. Higher prevalence (23.18%) was observed among women aged 20–29 compared to adolescents (4.1%). Only 21.9% of married women reported using modern contraceptives. At the individual-level, the factors that positively influenced use of modern contraceptives included: women’s marital status, wealth index and level of education while sex of the household head, ever terminated a pregnancy and religion negatively affected the use of modern contraceptives. At community-level, community access to health facilities was found to have negative influence on the use of modern contraceptives among women. In communities where women frequently visited health facilities in the 12 months preceding the survey, the use of modern contraceptives reduced by 3.9%. Accessibility analysis revealed challenges, with women in northeastern districts (rural districts) facing travel times exceeding four hours to reach health facilities.

**Conclusion:**

Utilization of modern contraceptives are essential for promoting women’s health and well-being, particularly concerning maternal healthcare. This study highlights disparities in modern contraceptive use across age groups and the districts, emphasizing the need for targeted interventions. Policymakers and stakeholders must prioritize strategies that promote utilization of modern contraceptives and maternal healthcare services to address these disparities effectively. Such efforts are crucial for improving reproductive health outcomes and reducing the burden of unintended pregnancies and related complications in Uganda.

## Background

Globally, the challenge of high rates of unintended and unwanted pregnancies persists despite ongoing efforts to enhance access to and utilization of contraceptive services, which are recognized as fundamental reproductive rights for women [1, 2]. In 2019, it was estimated that out of 1.11 billion women of reproductive age worldwide, 842 million use modern contraceptive methods for family planning, leaving around 270 million with unmet contraceptive needs [3]. Sub-Saharan Africa bears a significant burden of unintended pregnancies, with approximately 14 million cases recorded annually, a substantial proportion of which occur among adolescent women [4, 5]. Utilization of modern contraceptives is crucial in empowering women and their partners to effectively manage fertility, thereby reducing the incidence of unintended pregnancies, unsafe abortions, and pregnancy-related health complications and deaths [6–8]. Modern contraceptive methods encompass a wide range of options, including male and female condoms, oral contraceptives, injectables, implants, intrauterine devices (IUDs), and permanent methods such as female sterilization and vasectomy [9].

There are marked disparities in access and use of modern contraception across Sub- Saharan African countries among women of reproductive age. For instance, in a study conducted in 2021, in Chad, only 7.65% of women use modern contraceptives, the number slightly increases to 8.24% in DRC, and 11.38% in Angola, Ghana (20.74%), Sierra Leone (23.75%), Uganda (30.84%) while this figure rises to 62.23% in Zimbabwe [10]. Uganda has made concerted efforts to improve access to and utilization of contraceptive services, particularly through community-based distribution channels [11–13]. For instance, in 2012, Uganda committed to reducing the unmet need for family planning to 10% and increasing the prevalence rate of modern contraceptives to 50% by 2020 [13]. However, despite these efforts, the country has not achieved its targets for reducing unmet need for family planning and increasing modern contraceptive prevalence rates. In 2020, modern contraceptive prevalence rate in Uganda was 38.7% while modern family planning among married women was 30.5% [13]. Factors contributing to this discrepancy include limited access to modern contraceptives, distance to health facilities, inadequate skilled health personnel, fear of side effects, lack of male involvement, cultural beliefs, and limited awareness [14–17]. Therefore, conducting an assessment of the spatial distribution and factors associated with modern contraceptive utilization is essential for understanding the multifaceted factors contributing to this issue and for aligning efforts with national goals and Sustainable Development Goals (SDGs) [18, 19].

With the increasing availability of geographically referenced health facility and population data, conducting geospatial analyses has become feasible, allowing for the identification of areas with limited access to specific health services, such as family planning [20]. However, studies on the access and utilization of modern contraceptive use in Uganda have focused mainly on individual and overlooking the cluster levels and the potential geographic variations in access and utilization [19, 21]. In their study of spatial analysis of unmet need for family planning in Uganda, Baley et al. [19], found that high rate of unmet need (significant hotspots) was found in Northern part of Uganda, but did not assess the factors such as access to health facilities [19]. In their cross sectional study of access to family planning services in Uganda [22], only 176 health care facilities were surveyed [22]. Uganda’s decentralized health system, organized at the district level, aims to improve management and access to services, yet access to and utilization of modern contraceptives remain low across geographic areas [23]. Several studies have showed that the main barrier to utilization is access to health services [24–26]. Accessing and utilizing health care are separate but interconnected concepts that form the focal point of health policy and efforts to enhance quality [24]. Utilization presumes access and encompasses the development of a healthcare strategy during a health care encounter and its subsequent execution [24]. By identifying geographic disparities and associated factors influencing contraceptive utilization, this study seeks to inform targeted interventions and policy efforts aimed at improving access to and utilization of modern contraceptives, ultimately contributing to improved reproductive health outcomes for women in Uganda. This study aims to address these gaps by examining the prevalence, spatial distribution, and determinants of modern contraceptive utilization among women aged 15–49 years in Uganda.

## Methods

### Study area

**Fig. 1:**
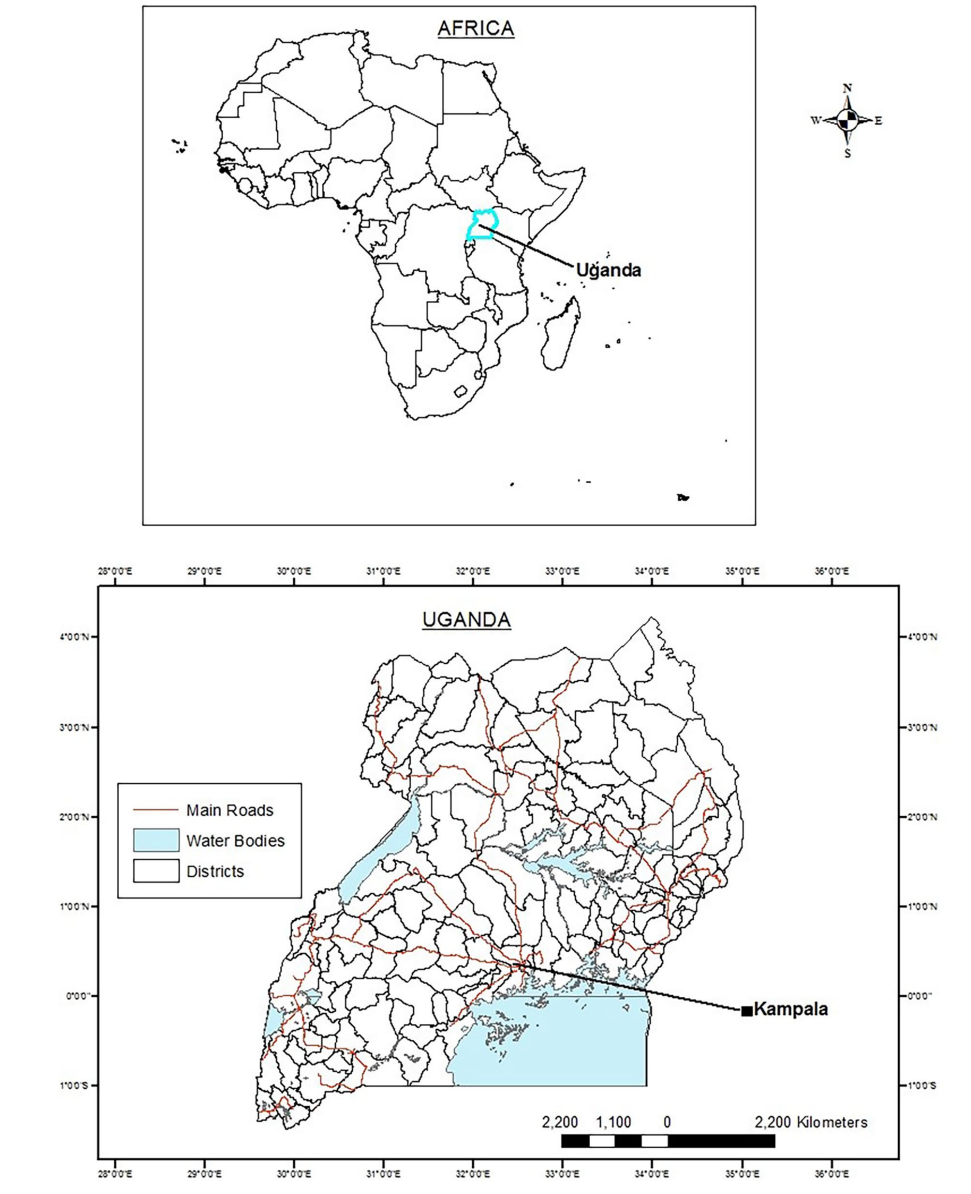
Locational map of Uganda

Uganda is a landlocked country located in southeast Africa between 1º S and 4º N latitude, and between 30º E and 35º E longitude (Fig. 1). It has diverse geographic features that include volcanic hills, mountains, and lakes. Although landlocked, Uganda contains many large lakes such as the world biggest Lake Victoria, other major ones such as Kyoga, Albert, Edward and the smaller one Lake George [27]. In terms of health and population, maternal health in rural Uganda lags behind national policy targets due to different factors such as geographical inaccessibility to health facilities, lack of transport and financial burdens identified as key demand-side constraints to accessing maternal health services [28]. There are disparities in the use of contraceptives among poor women (∼ 15%) and wealthy women (∼ 40%) which result in Ugandan women having about 6 children while they prefer to have approximately 4 children [29]. Uganda’s median age is 16 years, making it the lowest in the world (as of 2023), the country has also one of the highest (fifth) fertility rates in the world at about 5.5 children born per woman based on 2022 rates [30]. The road infrastructure which is the main barrier in terms of health care access is the most used (95% of freight and passenger traffic is handled by road traffic) in the country. The road network in Uganda is approximately 129,469 km (80,448 mi) long [31]. About 4% of these roads are paved which equates to only about 5,300 km (3,300 mi) of paved road [31].

### Data and methods

#### Study sample

**Fig. 2:**
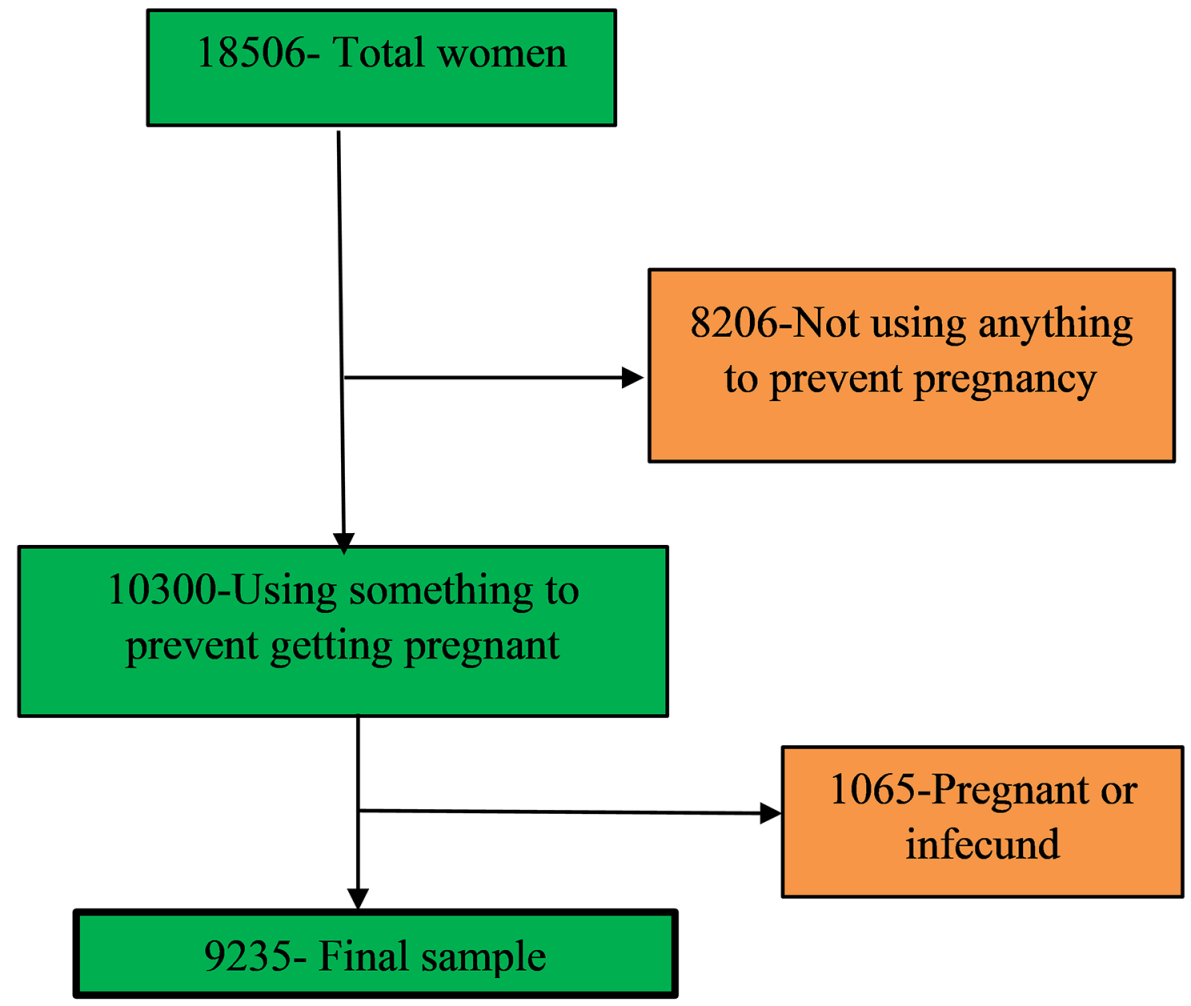
Women who participated in the study are depicted in the schematic. From the 18,506 women who took part in the 2016 Uganda Demographic and Health Survey, the final sample for this study was reached after using a number of stages or exclusion criteria. In the first stage, 8206 women who were not using any method to prevent pregnancy were excluded. Pregnant women were excluded based on the other criteria. Women, who claimed to have used any strategy to avoid becoming pregnant in the five years before to the survey, were included after a thorough screening. The final sample size of 9235 was selected

This study utilized secondary data from the 2016 Uganda Demographic and Health Survey (UDHS). Conducted through a two-stage stratified cluster sampling design, the 2016 UDHS incorporated rural-urban and regional components. The Uganda Bureau of Statistics provided a sampling frame based on the 2014 Uganda National Population and Housing Census (NPHC), resulting in 696 Enumerated Areas (EAs) sampled. Of the 20,791 households included in the sample, 19,938 were occupied, with successful interviews conducted in 19,588 occupied households, achieving a robust 98% response rate. Permission to access the dataset was obtained from the MEASURE International website (www.dhsprogram.com). The study focused mainly on a sample of 9,235 women aged 15 to 49 who reported to have used anything or tried in any way to delay or avoid getting pregnant. Further details regarding the study design and sampling methodology can be found in the 2016 UDHS report [14] and the accompanying flowchart (refer to Fig. 2).

#### Outcome variables

The dependent variable for this study was categorized as a dichotomous outcome: whether a woman used traditional or modern contraceptives. This variable was derived from a questionnaire administered to women, assessing whether they had ever utilized any method to delay or prevent pregnancy. If affirmative, methods such as female or male sterilization, IUD, injectables, implants, pills, condom, female condom, emergency contraception, and other modern methods were classified as modern contraceptives. Conversely, methods such as the rhythm method, withdrawal, lactational amenorrhea method, and other traditional methods were categorized as traditional methods [14].

#### Independent variables

The variables considered in the study include women’s age at survey, marital status, sex of the household head, number of children ever born, history of pregnancy termination, level of education, access to media, desire to have last child, wealth index, religion, and employment status. Maternal age at survey was categorized into four groups: 15–19, 20–29, 30–39, and 40+. Marital status encompasses single, married, cohabitating, or previously married (separated, divorced, and widowed) classifications. The sex of the household head is determined by individual who is recognized as household head by the household members, and may be male or female. Children ever born refer to the number of offspring a woman has had, categorized as 1, 2, 3, and 4 or more. History of pregnancy termination reflects whether women terminated pregnancies in the five years prior to the survey, categorized as yes or no. Education level is defined by the highest level of education attained by the mother: no education, primary, secondary, or higher. Level of education as provided for in the dataset and report ranged from no education, primary, secondary and higher education. However, due to fewer numbers of the respondents, we combined both categories of secondary and higher. This approach has been commonly used by other scholars who used DHS dataset as well [11]. Access to media is gauged by exposure to newspapers/magazines, radio, and television, classified as low, medium, or high. The media exposure variable was developed as a composite variable, from three forms of media: newspapers, magazines, radio, and television. The individual responses were based on frequency of access: 0 not at all, 1 less than once a week, and 2 at least once a week. These were recoded as individual responses into two categories, where 0 signified no access and 1–2 indicated access. The responses were computed from the variable, and the resulting sum ranged from 0 to 3. The median point was used, defining 0 as low, 1 as medium, and 2–3 as high access, due to the non-normal distribution of the sum [10]. Child wantedness pertains to whether a woman desired her most recent pregnancy: wanted then, wanted later, or wanted no more. Employment status distinguishes between women who were employed or unemployed in the preceding 12 months. Religion encompasses affiliations such as Anglican, Catholic, Muslim, and other religious groups. The wealth index, reflecting household socioeconomic status, is categorized as poor (poorest and poorer), middle, and rich (rich and richest).

#### Community-level variables

The community-level variables were derived by aggregating individual-level characteristics at the community (District) level. Aggregate variables were categorized as high or low based on the distribution of proportion values calculated for each district, with the exception of place of residence. The community-level variables examined in this study include: place of residence, community age group, community socioeconomic status, community access to family planning messages via media, community level of education, and community access to health facilities. Place of residence categorizes the type of dwelling where the woman was found during the survey night into rural or urban settings. Community age group was determined based on the age at survey and classified into two groups: young (women aged 15–29) and old (women aged 30–49). Community socioeconomic status was established from wealth index, with the poor representing low socioeconomic status and the high category including women from middle to richest categories. Community access to media on family planning is determined by whether women have at least one source of media for family planning information, such as newspapers, radio, or television, compared to those without access. Community level of education indicates the proportion of women with at least secondary education versus those with primary education or no education. Community access to health facilities represents the proportion of women who sought care at health facilities within the past twelve months. To further unpack utilization, access was measured using two variables (i) place of residence and (ii) Community access to health facilities.

### Data analysis

#### Statistical analysis

The study utilized the women’s records dataset to analyze secondary data from the UDHS 2016. Data analysis was performed using STATA version 14.2 [32]. Descriptive statistics, presented in percentages, were employed to summarize key variables in the univariate data. At the bivariate level, frequencies and cross-tabulations were conducted to examine the distribution of outcome variables by individual and community-level characteristics. Pearson’s chi-square test was employed to assess the relationship between individual and community-level characteristics. Categorical variables with *p*-values of less than 0.05 in the bivariate model underwent testing for multicollinearity using the Variance Inflation Factor (VIF), with a threshold set at 10; any variable with a VIF greater than 10 was removed from the model. Results indicate the absence of multicollinearity (mean VIF = 1.25, min VIF = 1.01, max VIF = 1.85), and these variables were included in the final multivariate model. The outcome variable was dichotomous, and a multilevel binary logistic regression model was utilized to examine the relationship between individual and contextual variables and the use of modern contraceptives. Fixed and random effects were calculated to evaluate individual and community variations. The fitted multilevel binary logistic regression model was referenced [33].

1 $$log\left[\frac{{\pi }_{ij}}{1-{\pi }_{ij}}\right]={\beta }_{0}+{\beta }_{1}{\mathcal{X}}_{1ij}+{\beta }_{2}{\mathcal{X}}_{2ij}+{\beta }_{n}{\mathcal{X}}_{nij}+{\mu }_{0j}$$

Where *π*_*ij*_ represents the probability of the i^th^ individual in the j^th^ community using modern contraceptives, *(1-π*_*ij*_) denotes the probability of the i^th^ individual in the j^th^ community not using modern contraceptives, β_0_ stands for the log odds of the intercept, and β_1_, … β_n_ represent the effect sizes of individual and community-level factors. X_1ij_… X_nij_ denote the independent variables at both individual and community levels, while u_Oj_ represents the quantities of random errors at cluster levels. Four multilevel binary logistic regression models are employed to assess the association between individual and community-level variables and the use of modern contraceptives. In the initial, empty model (Model 1), no covariate was included, allowing examination of the random influence of variability between districts. The inter-class correlation coefficient (ICC) was calculated to justify the multilevel analysis method by demonstrating the level of variance between districts. The second model (Model 2) determined the effects of individual-level characteristics on women’s use of modern contraceptives. The ICC was recalculated to observe any change in between-districts variability upon adding individual-level characteristics to the empty model. The third model (Model 3) introduced community-level characteristics while excluding individual-level characteristics. In the fourth model (Model 4), which is the combined model, both individual-level and community-level characteristics were included to display their net fixed and random effects. The random effect was explained using the inter-Class Correlation (ICC) formula: [ICC = σu^2^ / (σu^2^ + π^2^ /3)]. The fixed-effect sizes of individual-level and community-level factors on the use of modern contraceptives were stated using the marginal effect values. The statistical significance level was set at a *p*-value less than 0.05 [33]. The log-likelihood ratio tested the adequacy of the model, while the Akaike Information Criteria (AIC) assessed how well the different models fit the data [34].

#### Spatial analysis

To visualize the modern contraceptive percent use by women in Ugandan districts, choropleth mapping in ArcGIS 10.8.2 [35] was used through the Jenks method. The natural breaks (Jenks) classification method was utilized to categorize spatial data into distinct groups based on their inherent variation [36]. This approach is considered an optimization method as it aims to minimize within-class variation while maximizing between-class variation, thus facilitating the identification of meaningful and homogeneous clusters within the dataset. The process of natural breaks classification involves the application of algorithms designed to group the data into mutually exclusive and internally homogeneous classes. These algorithms seek to identify breakpoints or thresholds in the data distribution where transitions occur from one class to another, with each class exhibiting relatively low variability within and distinct differences from other classes [37]. Five classes were adopted with the smallest class at 0 including districts without data values and the highest class at 7.6%. To measure access (unpacking utilization) [24], spatial analysis was used through the Network analyst technique, in ArcGIS 10.8 [38]. The unit of analysis for the geographic results was Uganda’s national sub administration boundaries (districts). This was informed by the community variables (place of residence and community access to health facilities) that were individual variables aggregated to the district level. The district shapefiles were obtained from https://data.humdata.org/dataset/. Community access to health facilities represented the proportion of women who sought care at health facilities within the past twelve months, therefore only government facilities (*n* = 3190 out of all 3601 facilities in Uganda) were used for analysis. In the statistical analysis (Table 1), the variable –community access to health facilities was statistically significant which led to further spatial analysis. The facilities were not differentiated whether it was a clinic or a hospital. The road network was obtained from the same source and was classified into five road types (Table 2). The shapefiles were projected to the Arc 1960 UTM Zone 36s [39] where Uganda is located.

**Table 1:** Background characteristics and percentage of women who utilized Contraceptives by individual and community-level characteristics

Variables	Frequency *n* (%)	Traditional contraceptive (%)	Modern contraceptive (%)	Chi-square *P*-value
**Age at survey**				< 0.001
<=19	675 ( 7.4)	3.2	4.2	
20–29	3859 (42.0)	18.6	23.4	
30–39	3043 (32.6)	15.1	17.5	
40+	1661 (18.0)	9.7	8.1	
**Marital Status**				< 0.001
Single	979 (10.7)	5.3	5.4	
Married	3547 (37.0)	15.9	21.1	
Living together	3204 (35.2)	15.1	20.0	
Previous married	1508 (17.1)	10.4	6.7	
**Sex of household head**				< 0.001
Male	6217 (66.4)	28.1	38.4	
Female	3021 (33.6)	18.7	14.9	
**Children ever born**				0.081
1	1850 (22.0)	10.5	11.4	
2	1376 (16.4)	7.2	9.2	
3	1275 (15.4)	7.0	8.4	
4+	4737 (46.2)	22.0	24.2	
**Ever terminated pregnancy**				< 0.001
No	7152 (77.4)	35.0	42.4	
Yes	2086 (22.6)	11.9	10.8	
**Level of Education**				< 0.001
No education	849 (8.4)	4.5	3.9	
Primary	5355 (55.9)	26.1	29.8	
Secondary	3034 (35.7)	16.1	19.6	
**Media**				0.429
Low	2650 (26.9)	12.6	14.2	
Medium	3408 (35.4)	16.8	18.6	
High	3180 (37.7)	17.3	20.5	
**Wanted last child**				0.165
Wanted then	3432 (53.9)	23.18	30.7	
Wanted later	2129 (33.4)	14.5	19.0	
Wanted no more	824 (12.7)	6.2	6.5	
**Wealth Index**				0.031
Poor	3201 (30.6)	15.0	15.6	
Middle	1768 (18.6)	8.2	10.3	
Rich	4269 (50.8)	23.5	27.4	
**Religion**				0.100
Anglican	3035 (32.3)	14.5	17.9	
Catholic	3622 (38.6)	18.2	20.4	
Muslim	1125 (13.5)	6.4	7.1	
Other’s	1456 (15.6)	7.8	7.9	
**Employment Status**				0.744
Unemployed	1675 (19.2)	9.0	10.1	
Employed	7563 (80.8)	37.7	43.1	
**Place of Residence**				0.799
Urban	2460 (30.0)	14.1	15.9	
Rural	6778 (70.0)	32.7	37.4	
**Community Age Group**				0.827
Young	4754 (52.9)	24.8	28.1	
Old	4484 (47.1)	22.0	25.2	
**Community socio-economic status**				0.073
Low	1125 (9.4)	4.6	4.7	
High	8113 (90.6)	42.1	48.5	
**Community Media access to FP**				0.335
Low	5746 (57.3)	27.1	30.2	
High	3492 (42.7)	19.6	23.1	
**Community level of education**				0.214
Low	5621 (54.8)	26.1	28.7	
High	3617 (42.7)	20.7	24.5	
**Community access to health facility**				0.303
Low	4534 (47.3)	21.8	25.6	
High	4704 (52.7)	25.0	27.7	
**Total**	**9238**	**46.8**	**53.2**	

**Table 2:** Road network and level of modern contraceptive use among women in Uganda

Road Type	National Speed	Length of the road/Maximum speed	Assigned driving time (Minutes)	Modern contraceptive use	Traditional contraceptive use
Primary	120 km/h	0.5	30	4914(53.2%)	4324(46.8%)
Secondary	80 km/h	0.75	45		
Tertiary	60 km/h	1	60		
Residential	40 km/h	1.5	90		
Tracks	20 km/h	3	180		

From the road network, there were many different types of roads that included construction and service roads but only five were used in the network analysis because there had national driving speeds [28]. The detailed driving speed limit for all road types in Uganda are shown on Table 2. Access was assessed using road network analysis using Service area tool [40] in the Network analyst from ArcGIS 10.8 [35]. It was done in two steps, where the first step involved building a road network, to do so, the following formula was used [40, 41];

2 $$\begin{array}{l}{\rm{Driving}}\,{\rm{time}}\,{\rm{ = }}\\\frac{{length\,of\,the\,roads}}{{maximum\,speed\left( {for\,each\,road\,type} \right)}} * 60\end{array}$$

To build a road network, place of residence was vital because it categorized the type of dwelling where the woman was found during the survey night into rural or urban settings. With this information, it was easier to classify types of roads as well as the driving times based on where they were mostly located either (rural or urban). The assigned driving times assumed for this study were 30 min for primary roads (mostly passing through urban areas), 45 min secondary, 60 min tertiary roads, 90 min residential and 180 min for tracks (mostly passing through urban areas (Table 2). The second step involved modelling the driving times and to perform this task, the search tolerance was set at 15 km which is the estimated distance of the women’s nearest health facility. The WHO [42] recommends 5 km as the radius of the nearest health facility and in most cases this distance is increased to 10 km [43, 44] to cater for rural areas. In this study it was further increased to 15 km to cater for the areas in the lake regions. Water bodies (lakes) were added as restrictive barriers [41], predicting that women will use alternative routes to the health facilities to access contraceptives. The direction was set to drive towards the health facility. The output was generated with six generalized non-overlapping polygons. The sixth polygon (180–240 min) was added to further account for the lake areas that did not have any roads that were closest to the health facilities. From the 3,190 government health facilities that were used in the analysis, two were not located in the network (locations 2695 and 2803) resulting in 3,188 health facilities in the model.

## Results

### Descriptive characteristics

Table 1 presents the distribution of the study population. The largest age group among women was 20–29 years, comprising 42% of the sample, followed by those aged 30–39 years (32.6%). Adolescents (15–19 years) constituted the smallest category, accounting for 7.3% of the total. The majority of women were either married (37%) or cohabiting (35.2%), while single women comprised the smallest proportion (10.7%). Approximately two-thirds (66.4%) of the women belonged to households headed by males. Regarding family size, more than half (46.2%) of the women had four or more children, with only 22.6% reporting terminated pregnancies. In terms of education, the majority (55.9%) had received primary education, while only 35.7% had attained secondary or higher education. Above one-third (37.7%) of the women had high access to media. Regarding fertility desires, 53.9% of women desired children at that time, whereas 12.7% desired no more children. A significant proportion (50.8%) of women belonged to the rich wealth index bracket. The Anglican and Catholic denominations constituted the majority (32.3% and 38.6% respectively) of the population. Most of the women (80.8%) were reported to be employed, and the majority (70.0%) resided in rural areas. Additionally, more than half (52.9%) of the women were young, and the vast majority (90.6%) belonged to high socio-economic status communities. The majority (57.3%) belonged to communities with low access to family planning messages from the media, and a similar proportion (54.8%) came from communities with low levels of education. Finally, over half (52.7%) resided in communities with high access to health facilities.

### Prevalence of modern contraceptive use across the independent variables

Table 1 illustrates the percentage distribution of women according to modern contraceptive use and various background characteristics. Of the total sample, 4,914 women (53.2%) reported using modern contraceptives. Among the individual-level variables examined, the utilization of modern contraceptives varied significantly across age groups, marital status, sex of the household head, history of pregnancy termination, educational attainment, desire for last child, wealth index, and religious affiliation. Specifically, the prevalence of modern contraceptive use was higher (23.4%) among women aged 20–29 compared to adolescents aged 15–19 (4.2%). Only 21.1% of married women reported using modern contraceptives, followed by those in cohabiting relationships. A notable proportion (38.4%) of women from households headed by males used modern contraceptives compared to female-headed households (14.9%). Women who had not terminated a pregnancy were more likely (42.4%) to use modern contraceptives compared to those who had terminated a pregnancy (10.8%). Similarly, women with primary education had a higher prevalence (29.8%) of modern contraceptive use compared to those with no education and those with secondary/higher education (3.9% and 19.6% respectively). Among women who desired a child at the time of the survey, a significant proportion (30.7%) used modern contraceptives. Additionally, over a quarter (27.4%) of women from wealthier households utilized modern contraceptives compared to those from poorer households (15.6%). Women belonging to Catholic denominations had a slightly higher rate of modern contraceptive use (20.4%) compared to those of the Anglican faith (17.9%). Regarding community-level variables, nearly half (48.5%) of women from higher socioeconomic backgrounds reported using modern contraceptives compared to those from lower socioeconomic backgrounds. Finally, a similar proportion of women had access to health facilities (27.7%), regardless of their usage of modern contraceptives.

### Results of the fixed effects

The model fit for the four models was examined, and the one with the smallest AIC was chosen as the best fit for the data. Among the four models assessed, model 4, which exhibited the lowest AIC statistic, was selected. This model was adjusted for both individual and community-level factors, showing a strong fit to the data compared to the others. Based on the results (Table 3) at the individual-level, the factors that positively influenced use of modern contraceptives include: women’s marital status, wealth index and level of education while sex of the household head, ever terminated a pregnancy and religion negatively affected the use of modern contraceptives. Being married or cohabiting increases the use of modern contraceptives by 9% and 7.3% respectively (Table 3). Additionally, women having primary (5.4%) and secondary/higher (5.9%) education increased their use of modern contraceptives. Wealth status increases use of modern contraceptives from women of middle and rich wealth index brackets by 3.6% and 3.8% respectively. Female headed households (7.3%) and having terminated a pregnancy (3.9%) reduce modern contraceptives use. Moreover, belonging to Islamic and other Christian denominations reduces use of modern contraceptives by 5.8% and 3.9% respectively. At community-level, community access to health facilities was found to have negative influence on the use of modern contraceptives among women. In communities where women frequently visited health facilities in the 12 months preceding the survey, the use of modern contraceptives reduced by 3.9% (refer to Table 3).

**Table 3:** Marginal effects from multilevel logistic regression model on the use of modern contraceptives among women in Uganda, UDHS 2016

Variable	Model 1	Model 2	Model 3
	Individual-level	Community-level	Individual/Community-level
	dy/dx	dy/dx	dy/dx
**Age at survey**			
<=19 (Ref)			
20–29	-0.063(0.035)*		-0.064(0.035)*
30–39	-0.066(0.041)		-0.068(0.041)
40+	-0.053(0.045)		-0.056(0.045)
**Marital Status**			
Single (Ref)			
Married	0.084(0.034)**		0.090(0.034)**
Living together	0.072(0.032)**		0.072(0.032)**
Previous married	-0.011(0.039)		-0.10(0.039)
**Sex of household head**			
Male (Ref)			
Female	-0.073(0.0.018)***		-0.073(0.017)***
**Children ever born**			
1 (Ref)			
2	0.006(0.027)		0.005(0.027)
3	-0.009(0.028)		-0.010(0.028)
4+	0.007(0.030)		0.009(0.030)
**Ever terminated pregnancy**			
No (Ref)			
Yes	-0.040(0.017)**		0.039(0.017)**
**Level of Education**			
No education (Ref)			
Primary	0.055(0.027)**		0.054(0.027)**
Secondary	0.059(0.030)*		0.056(0.030)*
**Media**			
Low (Ref)			
Medium	-0.020(0.017)		-0.020(0.017)
High	0.000(0.020)		0.001(0.021)
**Wanted last child**			
Wanted then (Ref)			
Wanted later	0.009(0.015)		0.010(0.015)
Wanted no more	-0.026(0.025)		-0.026(0.025)
**Wealth Index**			
Poor (Ref)			
Middle	0.045(0.019)**		0.036(0.019)**
Rich	0.048(0.021)**		0.038(0.022)**
**Religion**			
Anglican (Ref)			
Catholic	-0.020(0.018)		-0.019(0.018)
Muslim	-0.055(0.026)**		-0.058(0.026)**
Other’s	-0.037(0.018)**		-0.039(0.018)**
**Employment Status**			
Unemployed (Ref)			
Employed	0.024 (0.017)		0.027(0.017)
**Place of Residence**			
Urban (Ref)			
Rural		0.004(0.018)	0.001(0.021)
**Community Age Group**			
Young (Ref)			
Old		0.006(0.019)	0.011(0.021)
**Community socio-economic status**			
Low (Ref)			
High		0.025(0.028)	0.028(0.030)
**Community Media access to FP**			
Low (Ref)			
High		0.004(0.024)	0.009(0.028)
**Community level of education**			
Low (Ref)			
High		0.015(0.025)	0.024(0.030)
**Community access to health facility**			
Low (Ref)			
High		-0.030(0.018)*	-0.038(0.021)*
**Random effect results**			
PSU Variance (95%CI)	0.08(0.04–0.13)	0.10(0.06–0.17)	0.07(0.86–1.28)
ICC	0.023	0.030	0.021
Wild chi-square and *p*-value	Ref	139.59, *p* < 0.000	10.53, *p* < 0.000
**Model fitness**			
Log-likelihood	-6357.67	-4276.47	-6352.37
AIC	12719.34	8596.94	12712.73
PSU	112	112	112
**N**	**6385**	**6385**	**6385**

Note: *n* = 9238; (Source: Uganda Demographic and health survey data 2016) Figures in parentheses are standard errors. ***, **, and * denote that the difference is statistically significant at the 1%, 5% and 10% levels, respectively

### Spatial analysis results: measuring access and utilization

**Fig. 3:**
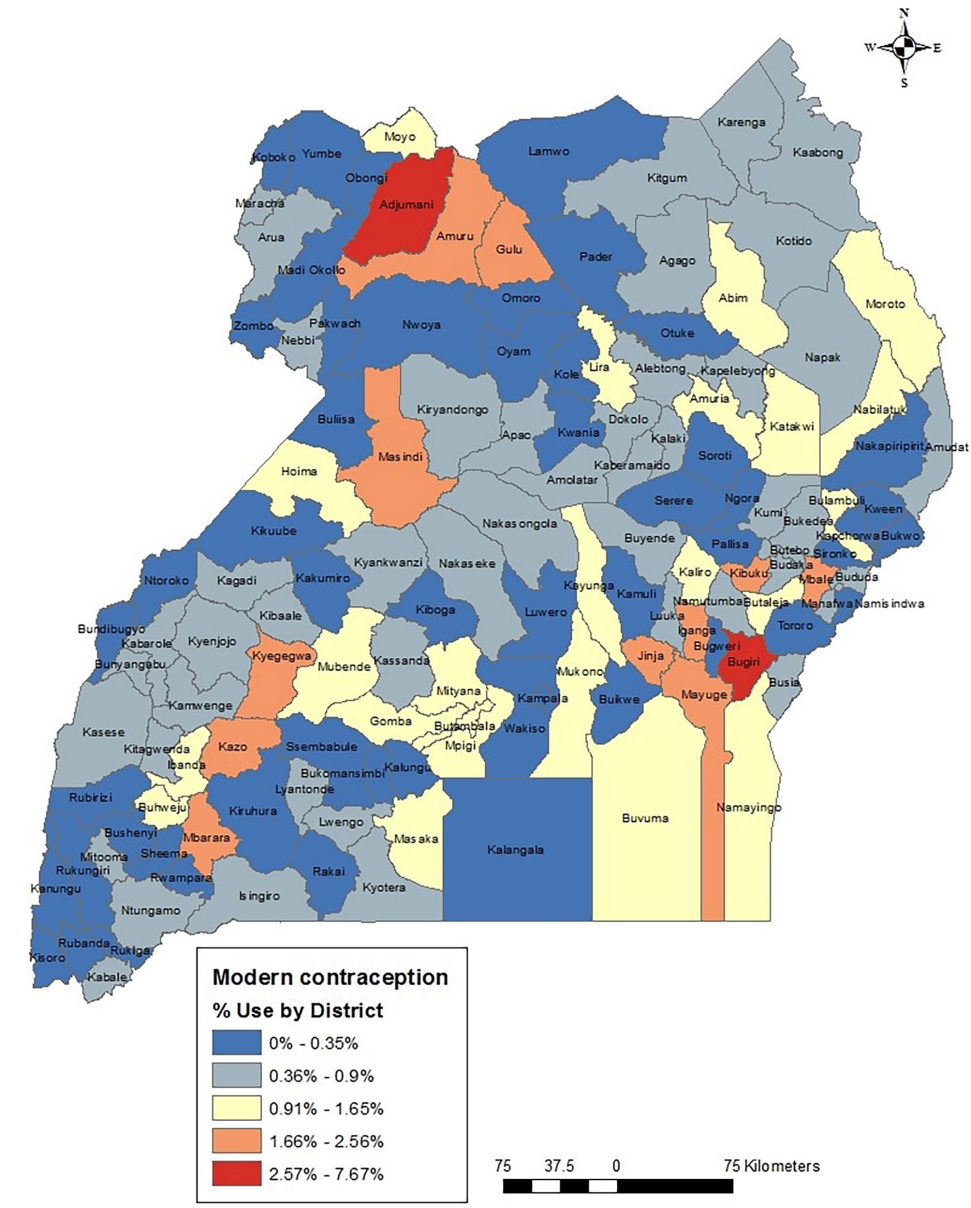
Modern contraceptive use by districts in Uganda, 2016

The highest modern contraceptive use was found out to be 7.67% (Fig. 3) among women in various districts of Uganda. There were two districts (Adjumani in the northwest and Bugiri in the south east) of the country exhibiting the highest contraceptive use. Kampala, which houses the country’s capital [urban district] was among those with the least use (0.36–0.9%). In general, low use was spatially concentrated in the northwest districts (Fig. 3).

**Fig. 4:**
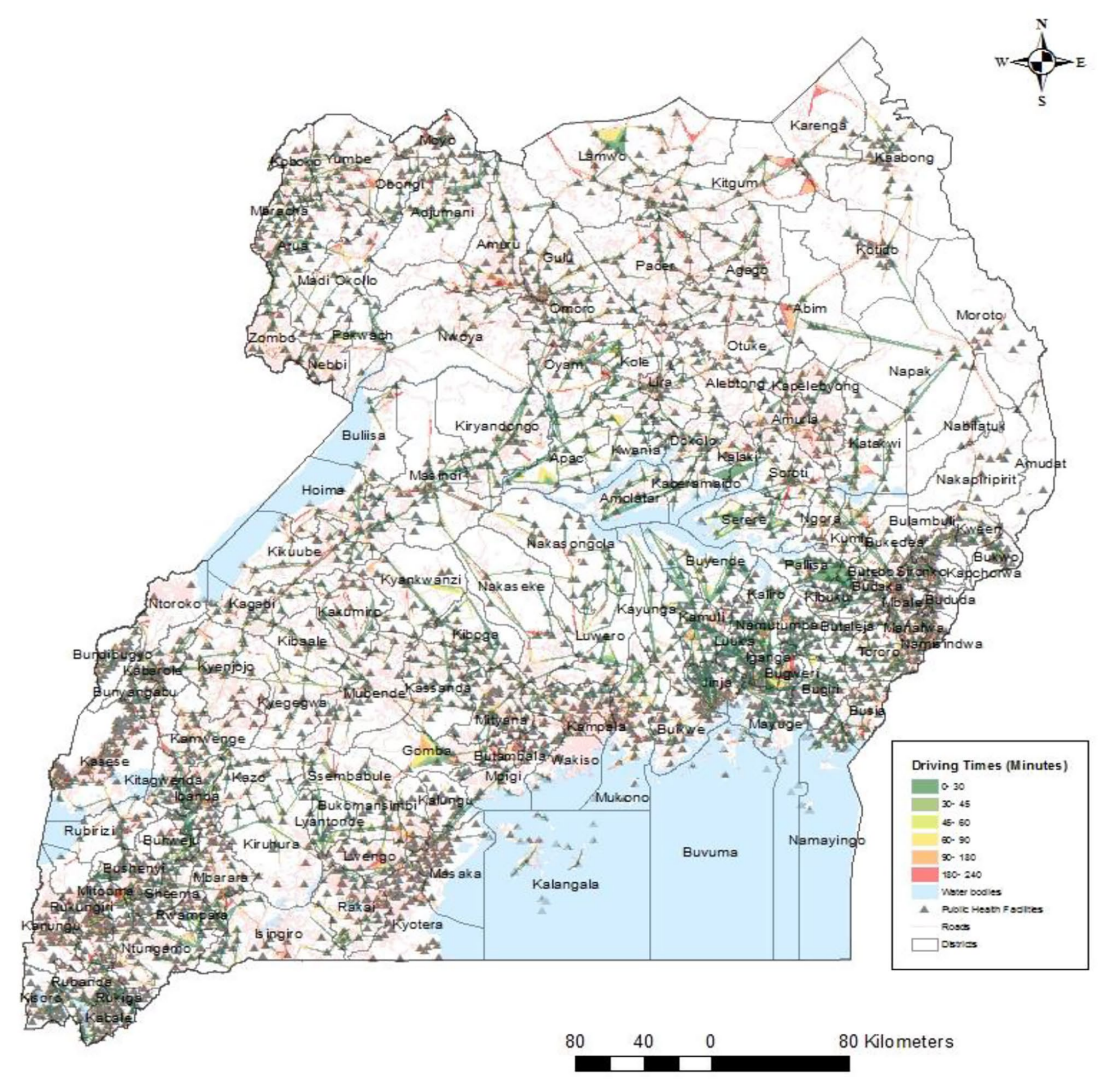
Driving times to public health centres in Ugandan Districts

**Fig. 5:**
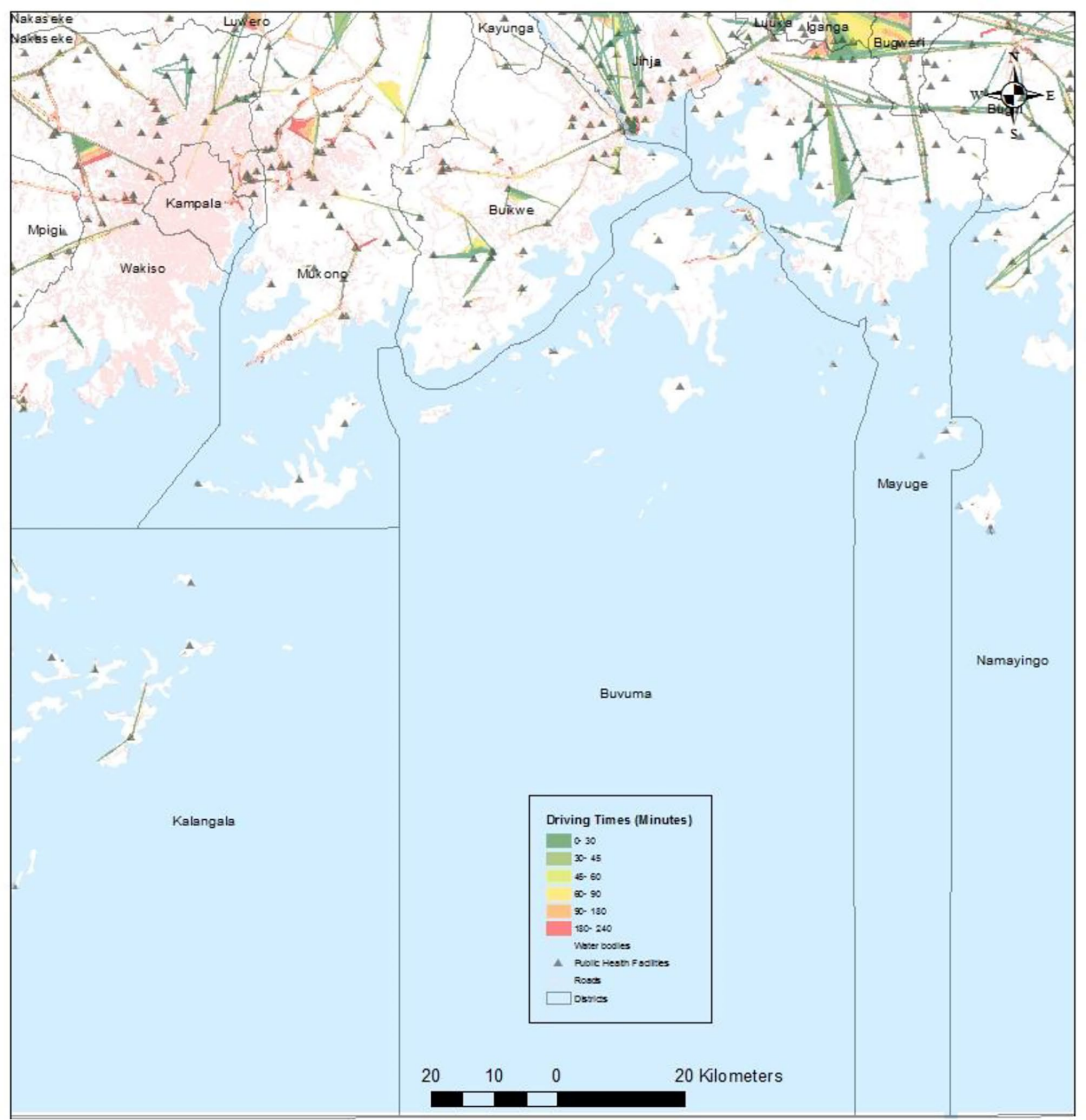
Insert map showing driving times to public health centres in the Lake regions

Figure 4 illustrates the travelling times in minutes for women to public health facilities. Many health facilities were outside the roads, particularly the main roads, making the road network to have many sharp edges. Many women are forced to travel more than four hours to reach the nearest health facility in the north eastern side of the country (rural districts) in districts such as Karenga and Abim (Fig. 4). There is a concentration of health centres that fall on the road network in the south east part (urban districts), which explains why most women will drive shortest distances of about 30 min (Fig. 4). A closer look shows that areas in the lake region have very limited geographic accessibility with a few roads and health facilities (Fig. 5). The two facilities that were not located in the network (facilities 2695 and 2803) were found in the lake area in Buvuma district (extreme south east). Even though Kampala is the capital of the country and has many health facilities, many women would have to drive at least minimum of 90 min (indicated by the orange colour) and maximum of 240 min (red colour) to reach the nearest health facility (Fig. 5). This is because most of these health facilities are not within the service area (close driving times).

## Discussion

Access and utilization to modern contraceptives and maternal healthcare services is not only a matter of health but also a fundamental human right, as recognized by various international instruments and agreements. This study underscores the critical role that access and utilization of modern contraceptives play in promoting health and well-being, particularly in the context of maternal health care. This study found two main findings; prevalence of modern contraceptives and determinants of modern contraceptive use.

### Prevalence of modern contraceptives

The study found that prevalence of modern contraceptive use was 53.19% (*n* = 4,919) in Uganda (Table 1). Modern contraceptive use showed even lower prevalence rates with spatial variation by districts where the highest level of use was 7.67% (Fig. 3). In Ghana, the spatial variation results showed prevalence of 13% [15]. In this current study, the northern region which is mainly rural, had high modern contraceptive use compared to the central region which is mostly urban. This finding is corroborated by studies in Uganda [11, 16]. It was contrasted by Paul et al. [17], who found that urban districts such as Kampala have high modern contraceptive use. Mankelkl et al. [6] also found that in Kenya, women in urban areas tend to use modern contraception compared to those in rural areas [6]. The identification of two districts exhibiting the highest modern contraceptive use, contrasted with the low prevalence observed in other regions, underscores the importance of considering spatial dynamics in reproductive health programming. Spatial disparities in contraceptive use may reflect variations in access to healthcare services, cultural norms, socioeconomic factors, and other contextual factors that influence reproductive health behaviors at the local level. Despite Kampala being a major urban center with relatively better access to healthcare services and information, the low contraceptive prevalence in Kampala suggests factors beyond mere access to services. Cultural beliefs, religious influences, and other sociocultural factors may influence contraceptive decision-making in urban settings, highlighting the need for targeted outreach and education efforts tailored to urban populations. A study by Makumbi et al. [45]. , in Uganda, found lower modern contraceptive prevalence of 34.2% among both men and women without taking into account location (rural or urban). The study included both men and women (*n* = 1346). Another Ugandan study by Daisy et al. [46], found modern contraceptive use to be 30.86% among 418 women recruited at a tertiary hospital. In Nigeria, modern contraceptive use level was found to be 43.7% among 400 women [47]. In this current study, the prevalence of modern contraceptive use was higher (23.18%) among women aged 20–29. This finding corroborates studies in Malawi [48], Ethiopia [49] and Bangladesh [50]. The observed higher prevalence of modern contraceptive use among women aged 20–29 underscores the importance of targeting reproductive health interventions towards this age group, which represents a critical stage in women’s reproductive lives. Efforts to promote contraceptive access and education among younger women could help prevent unintended pregnancies and reduce the risk of maternal mortality and morbidity associated with early or closely spaced pregnancies. A quarter of women from wealthier households utilized modern contraceptives compared to those from poorer households (18%). The disparities in modern contraceptive use based on household wealth highlight the influence of socioeconomic factors on access to reproductive health services. Women from wealthier households were found to have a higher prevalence of modern contraceptive use compared to those from poorer households. This suggests that economic barriers may pose significant challenges to contraceptive access for women from lower-income households. Addressing these disparities requires targeted interventions to improve access to affordable contraceptive services for disadvantaged populations.

### Determinants of modern contraceptives

Women from households headed by females were 25% less likely to use modern contraceptives compared to those from male-headed households. This finding aligns with Beyene et al. [51], in Ethiopia among 8,885 women of reproductive age. The association between household headship and contraceptive use further emphasizes the importance of gender dynamics in shaping reproductive health behaviors. Women from households headed by females were less likely to use modern contraceptives compared to those from male-headed households. This may reflect underlying gender inequalities and power dynamics within households, which can influence women’s autonomy and decision-making regarding reproductive health. Interventions aimed at promoting gender equity and women’s empowerment may help address barriers to contraceptive access and uptake among women in female-headed households. A study in Kenya [6] contrasted our results where they found that women from female headed households were 1.016 times more likely to use modern contraception. Additionally, women having primary and secondary/higher education increased their use of modern contraceptives by 5.4% and 5.9% respectively. Educational attainment emerged as a significant predictor of modern contraceptive use, with women having some form of education being more likely to use modern contraceptives compared to those with no education. This highlights the role of education in empowering women with knowledge and skills to make informed decisions about their reproductive health [52]. Investing in education, particularly for girls and young women, can have far-reaching benefits for improving reproductive health outcomes and reducing fertility rates. Furthermore, at the community level, in communities where women frequently visited health facilities in the 12 months preceding the survey, the use of modern contraceptives reduced by 3.9%. Looking at the spatial accessibility, the results showed there was a concentration of health centers that fell on the road network in the south east part, which could explains why most women will drive shortest distances of about 30 min (Fig. 4). Even though the women are not forced to drive longer hours, compared to some in north east districts (Karenga in Fig. 4), in terms of spatial distribution of use, the level of modern contraceptive use was less than 1% (Fig. 3). This findings mirror of a study done in Ethiopia where women in border far states were less likely to use contraceptives [53]. At the community level, the negative association between frequent visits to health facilities and modern contraceptive use raises important considerations regarding the quality and accessibility of contraceptive services. Women in communities with frequent health facility visits were less likely to use modern contraceptives compared to those who did not frequent health facilities. This may indicate barriers to contraceptive access or quality of care within health facilities, including stock outs of contraceptive supplies [54], provider bias [55], or inadequate counseling services. Strengthening health systems and ensuring the availability of high-quality contraceptive services at the community level are essential for addressing these barriers and promoting modern contraceptive uptake and utilization.

### Study limitations

Notable limitations of this study are (i) social desirability bias as most of the independent variables were self-reported and could not be verified through cross sectional studies. Also, explanatory variables such as residence, education and wealth reflected the women’s conditions at the time of the survey and not at the time of childbirth and hence women may have moved from one category of classification into another. However, secondary data used in this study was collected through the robust validated DHS surveys. (ii) DHS data is collected at the cluster level, when aggregating to the district level to perform the spatial analysis, it usually masks the important findings and (iii) health facilities were not differentiated by whether it was a clinic or hospital which women accessed modern contraceptives from. Hospitals as a higher hierarchy of health care, are usually well equipped which may lead to overestimation of modern contraceptive use, potentially influencing the accuracy of utilization.

## Conclusions

The findings of this study affirm that ensuring access and utilization to modern contraceptives is essential for empowering women, to make informed choices about their reproductive health. By providing individuals with the means to plan and space pregnancies, modern contraceptives contribute to reducing maternal mortality and morbidity, as well as improving overall maternal and child health outcomes. Moreover, the recognition of access to maternal health care as a fundamental human right, as stipulated by the WHO, underscores the importance of prioritizing efforts to ensure universal access to quality maternal healthcare services. This includes not only access and utilization of modern contraceptives but also comprehensive antenatal care, skilled attendance at birth, and postnatal care, among other essential services. It is imperative for policymakers, healthcare providers, and stakeholders to continue advocating for policies and programs that integrate geographic accessibility to modern contraceptives and maternal healthcare services. This requires a multi-faceted approach that addresses barriers to access and utilization, such as poor road networks, socio-economic factors, while also investing in healthcare infrastructure, workforce capacity, and community outreach initiatives.

## Data Availability

No datasets were generated or analysed during the current study.
